# Acceleration of opportunistic atrial fibrillation screening for elderly patients in routine primary care

**DOI:** 10.1371/journal.pone.0244240

**Published:** 2020-12-30

**Authors:** Akifumi Suzuki, Tomonori Okamura, Masahiro Sasaki, Hitoshi Matsuoka, Yoshinobu Ikeda, Akira Takahashi, Sayako Akiyama, Fumiko Ono, Nariaki Yoshihara, The Akita Study group

**Affiliations:** 1 Local Independent Administrative Institution, Akita Prefectural Hospital Organization, Akita, Japan; 2 The Japan Stroke Association, Osaka, Japan; 3 Department of Preventive Medicine and Public Health, Keio University School of Medicine, Tokyo, Japan; 4 Akita Cerebrospinal and Cardiovascular Center, Akita, Japan; 5 Matsuoka Internal Medicine Clinic, Akita, Japan; 6 Ikeda Clinic, Akita, Japan; 7 Takahashi Internal Medicine Clinic, Akita, Japan; 8 Market Access, Bayer Yakuhin, Ltd., Tokyo, Japan; 9 11 Primary Care Clinics in Daisen and Yokote, Akita, Japan; International University of Health and Welfare, School of Medicine, JAPAN

## Abstract

Cardio-embolic ischemic stroke caused by atrial fibrillation is more severe compared with other types of stroke, such as lacunar infarction and atherothrombotic infarction in patients without atrial fibrillation. Therefore, it is important to prevent cardio-embolic ischemic stroke by detecting atrial fibrillation early in at-risk patients and administering appropriate anticoagulation therapy. This prospective observational study aimed to evaluate the effectiveness of opportunistic atrial fibrillation screening at 12 primary clinics in Japan. The study included a 12-month pre-campaign period and a 12-month campaign period. During the campaign period, an awareness campaign was conducted to encourage physicians to be mindful of screening patients aged ≥65 years for atrial fibrillation by checking their pulses and performing subsequent electrocardiography when an irregular pulse was detected. The primary outcome was the proportion of patients with newly diagnosed atrial fibrillation. A sub-analysis focusing on first-time outpatients was performed. There were 9921 and 10,282 patients with no history of atrial fibrillation in the pre-campaign and campaign periods, respectively. In the whole population, the proportion of patients with newly diagnosed atrial fibrillation was 0.9% throughout the pre-campaign and campaign periods. In the sub-analysis limited to first-time outpatients, the detection proportion increased from 1.6% to 1.9% during the campaign period. In terms of age stratification, a large increase in detection was observed, especially among patients aged 65–74 years (detection increased from 0.9% to 1.5%) and ≥85 years (detection increased from 2.9% to 3.3%) during the campaign period. Our findings suggest the feasibility of opportunistic atrial fibrillation screening in routine primary care practice in Japan. Of note, our findings suggest that opportunistic atrial fibrillation screening targeting first-time outpatients may be of clinical value.

## Introduction

A systematic review of 184 worldwide population-based studies estimated that the number of individuals with atrial fibrillation (AF) in 2010 was 33.5 million [1], and future projections predict at least a doubling of AF cases by 2050 [2]. In Japan, an epidemiological study reported that the prevalence of AF increases with age, particularly in those aged >65 years [3, 4]. In addition, age >75 years is considered one of the risk factors for ischemic stroke in patients with AF [5], while a previous study reported that age >65 years is a risk factor for ischemic stroke [3, 6]. The prevalence of ischemic stroke is higher in males compared with females in their seventies (3.44% vs. 1.12%, respectively) and eighties (4.43% vs. 2.19%, respectively) [7]. Approximately 0.8 million people have AF, with an estimated 1 million patients expected to have AF in 2030, given the continuous growth of the aging population [7].

Patients with AF have an approximately five-fold higher risk of stroke compared with patients without AF [8]. Furthermore, cardio-embolic ischemic stroke caused by AF is more severe than non-AF-associated stroke, such as lacunar infarction and atherothrombotic infarction [9]. Thus, early detection of AF in at-risk patients and appropriate oral anticoagulant treatment are extremely important to prevent cardio-embolic ischemic stroke. In Japan, a large-scale, community-based prospective survey of patients with AF reported that almost half of patients with AF had paroxysmal AF, and almost 37.5% of patients with paroxysmal AF were asymptomatic, while almost 67% of patients had persistent/permanent AF [10, 11]. Considering the profile of patients with AF, effective measures to detect new-onset AF in older patients are necessary to manage AF treatment and stroke prevention.

European Society of Cardiology guidelines recommend opportunistic AF screening in patients >65 years of age [12]. Opportunistic AF screening involves checking the pulse. If the pulse is irregular, electrocardiography (ECG) is performed, regardless of the purpose of the hospital visit. Opportunistic AF screening for patients aged ≥65 years is considered effective for an early AF diagnosis based on the results of several studies. Fitzmaurice et al. performed opportunistic AF screening over 12 months in patients aged ≥65 years and found that the proportion of patients with newly diagnosed AF increased significantly from 1.04% to 1.64% [13]. They also studied the effectiveness of systematic screening (invitation for ECG) in the same period. The proportion of patients with newly diagnosed AF increased significantly from 1.04% to 1.62%, and they concluded that the preferred method of screening in patients aged ≥65 years in primary care was opportunistic pulse taking with follow-up ECG. Other studies conducted in Ireland [14], the UK, [13, 15], and Denmark [16] also found opportunistic AF screening to be an effective and feasible method for the detection of new AF cases among patients aged ≥65 years. However, whether opportunistic AF screening is effective in Japan is yet to be determined. Therefore, the objective of this study was to investigate the effectiveness of opportunistic AF screening for patients in routine primary care practice in Japan.

## Materials and methods

### Study setting and design

**Fig 1:**
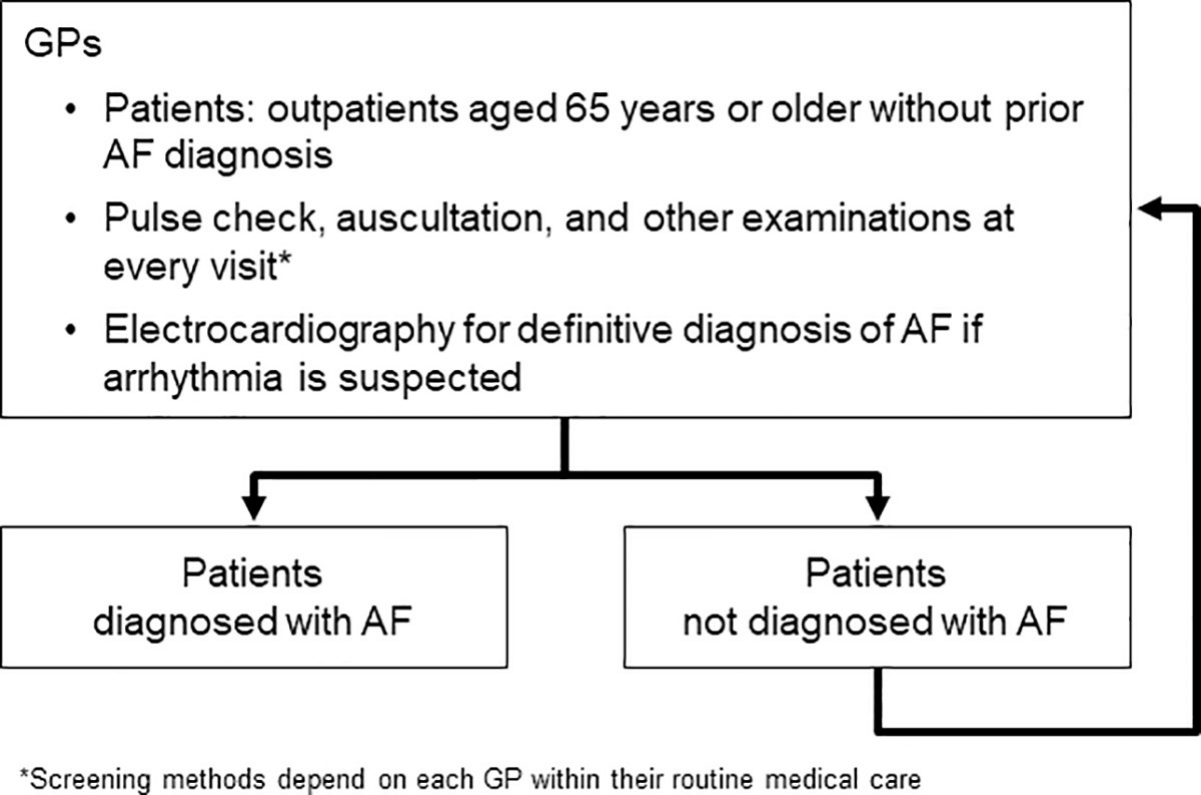
Flow of opportunistic AF screening. AF, atrial fibrillation; GP, general practitioner.

This study was conducted at a total of 12 primary care clinics in Daisen and Yokote, Akita Prefecture, Japan. All physicians agreed to participate in the study. The study consisted of two observational periods: a 12-month pre-campaign period (from October 19^th^, 2014 to October 18^th^, 2015) and a 12-month campaign period (from October 19^th^, 2016 to October 18^th^, 2017). The same physicians were involved in both periods. During the pre-campaign period, physicians performed routine medical examinations, including checking the pulse by palpation and/or auscultation as a standardized procedure. The campaign period was started following a strong recommendation for AF screening in usual primary care by our research committee. The campaign was directly targeted to physicians who volunteered to participate and consisted of an introductory meeting in which the research committee recommended that all physicians carefully perform opportunistic AF screening in usual primary care for all patients aged ≥65 years, irrespective of the type of primary disease (**Fig 1**).

Although pulse checking by palpation and/or auscultation is included in basic clinical practice in primary care in Japan, performing subsequent ECG after an irregular pulse is detected is not mandatory. However, in the present study, when an arrhythmia was suspected after checking the pulse, ECG was performed. Some clinics use automated sphygmomanometers with an irregular pulse detection function when measuring blood pressure. In the present study, the same automated sphygmomanometers with irregular pulse detection (HBP-1300/Fukuda Denshi Co., Ltd.) were provided to nine clinics by Bayer Yakuhin, Ltd., at their request. In Japan, automated sphygmomanometers are generally used in clinics and hospitals. These devices are available to physicians who are experienced in how to operate them. The fee for palpation, auscultation, and blood pressure measurement is included in the basic fee for primary care, and ECG for patients with suspected arrhythmia is also covered by the usual public medical insurance system in Japan (**S1 Text**). This study was conducted under the health insurance system in Japan, which consists of two main components: the universal health insurance system and free access (**S1 Text**).

Regarding the diagnosis of AF, primary care physicians who had access to ECG equipment in their clinics diagnosed AF on their own or referred the patient to a cardiologist if they were unsure of the diagnosis. Physicians without access to ECG equipment referred patients to a cardiologist for a definitive AF diagnosis.

### Data collection

Medical insurance claims data (**S1 Text**) of the study population were used to calculate the proportion of patients with newly diagnosed AF. Claims data were anonymized in each study clinic using a common application. Anonymous claims data were then sent to Medical Data Vision Co., Ltd., which is a consulting company that specializes in medical integration systems. Medical Data Vision generated a study patient ID, which was linked to the anonymous data from each clinic.

Data of outpatients aged ≥65 years who visited the study clinics during the pre-campaign or campaign periods were used. Data of outpatients previously diagnosed with AF at the starting point of each period were excluded. Patients with newly diagnosed AF were defined as outpatients diagnosed with AF for the first time at any study clinic during the pre-campaign or campaign periods by reviewing claims data.

### Statistical analyses

No formal hypothesis testing was performed for this study. Baseline characteristics of the study population eligible for AF screening were descriptively analyzed in the pre-campaign and campaign periods. The proportion of patients with newly diagnosed AF and 95% confidence intervals (CIs) in the pre-campaign and campaign periods were calculated. The proportion of patients with newly diagnosed AF was calculated as follows:

$$\frac{No.\;of\;patients\;with\;newly\;diagnosed\;AF\;aged\geq 65\;years\;}{No.\;of\;patients\;aged\geq 65\;years-No.\;of\;patients\;diagnosed\;with\;AF\;at\;the\;start\;}$$

The proportion of patients with newly diagnosed AF was compared between the pre-campaign and campaign periods in the whole target population and by sub-groups according to patient characteristics. The relative differences and relative ratios, along with their 95% CIs, were estimated. The relative differences and relative ratios of the proportion of patients with newly diagnosed AF were calculated in the pre-campaign period.

Furthermore, as an *ad hoc* analysis, patient characteristics and the proportion of patients with newly diagnosed AF were assessed in the subgroup of outpatients who visited the study clinics for the first time during the pre-campaign or campaign periods, and compared with the subgroup of returning outpatients (defined as patients who had visited a doctor at least one time before the start of the pre-campaign and campaign periods, and who continued to visit a doctor during the pre-campaign or campaign period). The two subgroups were likely to have different treatment records at each clinic due to cumulative visits; further, returning outpatients may have undergone various interventions, which may have affected the results of this study.

All statistical analyses were performed by I’cros Japan Co., Ltd. using SAS v9.4 (SAS Institute Inc., Cary, NC, USA).

### Ethical considerations

Because screening for arrhythmia should be conducted as standard care in primary care practice in Japan, individual informed consent for the screening procedure was not obtained. However, patients were informed about the study concept, including use of anonymous data for analysis, through *ad-hoc* posters placed in study clinics. The study protocol was collectively approved by the institutional ethics committee/institutional review board of the Research Institute for Brain and Blood Vessels, Akita, on behalf of all participating clinics (approval number: 15–10, approved on 5^th^ October, 2015) and the clinical research promotion network, Osaka, Japan (approved on 27^th^ August, 2015).

### Results

During the pre-campaign period, 10,583 patients aged ≥65 years underwent outpatient medical examinations. Of these, 662 patients had been previously diagnosed with AF at the start of the pre-campaign period or at the first visit. Therefore, 9921 patients were included in the study during the pre-campaign period. During the campaign period, 10,973 patients aged ≥65 years underwent outpatient medical examinations. Of these, 691 patients had been previously diagnosed with AF at the start of the campaign period or at the first visit. Therefore, 10,282 patients were included in the study during the campaign period.

Patient characteristics are shown in **Table 1**, and the proportion of patients with newly diagnosed AF during the pre-campaign and campaign periods is shown in **Table 2**. During the pre-campaign period, 86 patients had newly diagnosed AF, and the proportion of patients with newly diagnosed AF was 0.9% (86/9921). During the campaign period, 90 patients had newly diagnosed AF, and the proportion of patients with newly diagnosed AF was 0.9% (90/10,282). No difference in the proportion of patients with newly diagnosed AF was observed between the pre-campaign and campaign periods. However, the proportion of patients with newly diagnosed AF tended to be higher among male patients, patients aged ≥85 years, and patients with congestive heart failure during the campaign period compared with the pre-campaign period.

**Table 1:** Patient characteristics during the pre-campaign and campaign periods.

	Pre-campaign period	Campaign period
	(N = 9921)	(N = 10,282)
Male (n, %)	3622 (36.5)	3741 (36.4)
Age, years (n, %)		
65–74	4000 (40.3)	4178 (40.6)
75–84	3872 (39.0)	3857 (37.5)
≥85	2049 (20.7)	2247 (21.9)
Comorbidities (n, %)		
Congestive heart failure	1943 (19.6)	2098 (20.4)
Hypertension	6488 (65.4)	6761 (65.8)
Diabetes	3409 (34.4)	3837 (37.3)
History of stroke/TIA	897 (9.0)	915 (8.9)
Ischemic heart disease	1694 (17.1)	1891 (18.4)
Any comorbidity	7759 (78.2)	8146 (79.2)
CHA_2_DS_2_-VASc Score		
Median (Q1, Q3)	2 (1, 3)	2 (1, 3)

Abbreviations: CHA_2_DS_2_-VASc, congestive heart failure, hypertension, age, diabetes, previous stroke/transient ischemic attack–vascular disease; Q, quarter; TIA, transient ischemic attack.

**Table 2:** Proportion of patients with newly diagnosed AF during the pre-campaign and campaign periods.

	Proportion of patients with newly diagnosed AF (%)			
	Pre-campaign period	Campaign period	Relative difference (95% CI)	Relative ratio (95% CI)
**Total**	0.9 (86/9921)	0.9 (90/10,282)	0.0 (−0.2, 0.3)	1.0 (0.8, 1.4)
**Sex**				
Male	0.9 (32/3622)	1.1 (40/3741)	0.2 (−0.3, 0.6)	1.2 (0.8, 1.9)
Female	0.9 (54/6299)	0.8 (50/6541)	−0.1 (−0.4, 0.2)	0.9 (0.6, 1.3)
**Age, years**				
65–74	0.5 (21/4000)	0.4 (18/4178)	−0.1 (−0.4, 0.2)	0.8 (0.4, 1.5)
75–84	1.0 (38/3872)	1.0 (37/3857)	−0.0 (−0.5, 0.4)	1.0 (0.6, 1.5)
≥85	1.3 (27/2049)	1.6 (35/2247)	0.2 (−0.5, 1.0)	1.2 (0.7, 1.9)
**In-patients with comorbidities**				
Congestive heart failure	2.2 (42/1943)	2.5 (52/2098)	0.3 (−0.6, 1.2)	1.1 (0.8, 1.7)
Hypertension	1.0 (65/6488)	1.1 (71/6761)	0.0 (−0.3, 0.4)	1.0 (0.8, 1.5)
Diabetes	1.1 (39/3409)	0.9 (33/3837)	−0.3 (−0.7, 0.2)	0.8 (0.5, 1.2)
History of stroke/TIA	1.1 (10/897)	1.2 (11/915)	0.1 (−0.9, 1.1)	1.1 (0.5, 2.5)
Ischemic heart disease	1.4 (24/1694)	1.4 (27/1891)	0.0 (−0.8, 0.8)	1.0 (0.6, 1.7)
Any comorbidity	1.0 (81/7759)	1.1 (87/8146)	0.0 (−0.3, 0.3)	1.0 (0.8, 1.4)

Abbreviations: AF, atrial fibrillation; CI, confidence interval; TIA, transient ischemic attack.

Most patients were returning outpatients during both observational periods. There were 1720 first-time outpatients and 8201 returning outpatients during the pre-campaign period, and 1895 first-time outpatients and 8387 returning outpatients during the campaign period. The characteristics of first-time outpatients and returning outpatients during the pre-campaign and campaign periods are shown in **Table 3**. The distribution of sex and age was similar between groups. However, the proportion of patients with each comorbidity tended to be at least twice as high among returning outpatients compared with first-time outpatients in both observational periods.

**Table 3:** Characteristics of first-time/returning outpatients.

	Pre-campaign period (N = 9921)		Campaign period (N = 10,282)	
	First-time outpatients	Returning outpatients	First-time outpatients	Returning outpatients
	(N = 1720)	(N = 8201)	(N = 1621)	(N = 8661)
Male (n, %)	722 (42.0)	2900 (35.4)	647 (39.9)	3094 (35.7)
Age, years (n, %)				
65–74	820 (47.7)	3180 (38.8)	679 (41.9)	3499 (40.4)
75–84	592 (34.4)	3280 (40.0)	574 (35.4)	3283 (37.9)
≥85	308 (17.9)	1741 (21.2)	368 (22.7)	1879 (21.7)
Comorbidities (n, %)				
Congestive heart failure	182 (10.6)	1761 (21.5)	179 (11.0)	1919 (22.2)
Hypertension	483 (28.1)	6005 (73.2)	527 (32.5)	6234 (72.0)
Diabetes	266 (15.5)	3143 (38.3)	295 (18.2)	3542 (40.9)
History of stroke/TIA	82 (4.8)	815 (9.9)	70 (4.3)	845 (9.8)
Ischemic heart disease	160 (9.3)	1534 (18.7)	126 (7.8)	1765 (20.4)
Any comorbidity	744 (43.3)	7015 (85.5)	756 (46.6)	7390 (85.3)
CHA_2_DS_2_-VASc Score				
Median (Q1, Q3)	1 (0, 2)	2 (1, 3)	1 (0, 2)	2 (1, 3)

Abbreviations: CHA_2_DS_2_-VASc, congestive heart failure, hypertension, age, diabetes, previous stroke/transient ischemic attack–vascular disease; Q, quarter; TIA, transient ischemic attack.

The proportion of patients with newly diagnosed AF during the pre-campaign and campaign periods of first-time outpatients is shown in **Table 4**. Among first-time outpatients, the proportion of patients with newly diagnosed AF was 1.6% (27/1720) during the pre-campaign period and 1.9% (30/1621) during the campaign period. The proportion of patients with newly diagnosed AF tended to be higher among male patients, patients aged 65–74 years, patients aged ≥85 years, and patients with congestive heart failure, diabetes, history of stroke/transient ischemic attack, or ischemic heart disease during the campaign period compared with the pre-campaign period. Among patients aged 75–84 years, the proportion of patients with newly diagnosed AF decreased among first-time outpatients (1.9% in the pre-campaign period vs. 1.4% in the campaign period).

**Table 4:** Proportion of patients with newly diagnosed AF during the pre-campaign and campaign periods among first-time outpatients.

	Proportion of patients with newly diagnosed AF (%)			
	Pre-campaign period	Campaign period	Relative difference (95% CI)	Relative ratio (95% CI)
Total	1.6 (27/1720)	1.9 (30/1621)	0.3 (−0.6, 1.2)	1.2 (0.7, 2.0)
Sex				
Male	1.4 (10/722)	2.2 (14/647)	0.8 (−0.6, 2.2)	1.6 (0.7, 3.5)
Female	1.7 (17/998)	1.6 (16/974)	−0.1 (−1.2, 1.1)	1.0 (0.5, 1.9)
Age, years				
65–74	0.9 (7/820)	1.5 (10/679)	0.6 (−0.5, 1.7)	1.7 (0.7, 4.5)
75–84	1.9 (11/592)	1.4 (8/574)	−0.5 (−1.9, 1.0)	0.8 (0.3, 1.9)
≥85	2.9 (9/308)	3.3 (12/368)	0.3 (−2.3, 3.0)	1.1 (0.5, 2.6)
**In-patients with comorbidities**				
Congestive heart failure	7.1 (13/182)	8.9 (16/179)	1.8 (−3.8, 7.4)	1.3 (0.6, 2.5)
Hypertension	3.5 (17/483)	3.4 (18/527)	−0.1 (−2.4, 2.2)	1.0 (0.5, 1.9)
Diabetes	3.4 (9/266)	4.4 (13/295)	1.0 (−2.2, 4.2)	1.3 (0.6, 3.0)
History of stroke/TIA	3.7 (3/82)	7.1 (5/70)	3.5 (−3.8, 10.8)	2.0 (0.5, 7.9)
Ischemic heart disease	3.1 (5/160)	4.0 (5/126)	0.8 (−3.5, 5.2)	1.3 (0.4, 4.3)
Any comorbidity	3.4 (25/744)	3.7 (28/756)	0.3 (−1.5, 2.2)	1.1 (0.6, 1.9)

Abbreviations: AF, atrial fibrillation; CI, confidence interval; TIA, transient ischemic attack.

Among returning outpatients, the proportion of patients with newly diagnosed AF was 0.7% (59/8201) during the pre-campaign period and 0.7% (60/8661) during the campaign period (**S1 Table**). The proportion of patients with newly diagnosed AF did not change between the pre-campaign and campaign periods. This tendency was consistent with the subgroups of comorbidities and patient demographics.

## Discussion

Opportunistic AF screening is an effective and feasible method for the detection of new AF cases among patients aged ≥65 years [13–16]. Results of a systematic review and cost-effectiveness analysis of screening strategies for patients with AF aged ≥65 years showed that systematic opportunistic screening may be more cost-effective compared with systematic population screening [17]. In addition, patients with asymptomatic AF may further benefit from opportunistic AF screening, given that these patients are often not diagnosed until they present with an ischemic stroke event [18]. While the implementation of opportunistic AF screening among patients aged ≥65 years is supported as a cost-effective strategy in previous studies conducted in non-Asian patients, data from Japan are lacking.

Take Action for StroKe prevention in AF (TASK-AF) is a stroke prevention and advocacy project that is developing, piloting, and promoting recommendations aimed at reducing the burden of AF-related stroke [19]. This project is jointly run by the Japan Stroke Association and Bayer Yakuhin, Ltd. Two studies are ongoing under this project to obtain empirical evidence for substantiating the project’s recommendations [19]. As part of this project, this study aimed to investigate the effectiveness of opportunistic AF screening among patients aged ≥65 years in a primary care setting in Japan.

We observed no notable difference in the proportion of patients with newly diagnosed AF after implementation of opportunistic AF screening. This result differs from previous studies. Fitzmaurice et al. showed that the proportion of patients with newly diagnosed AF increased from 1.04% to 1.64% with opportunistic AF screening [13] and from 1.04% to 1.62% with systematic screening. Lowers et al. reviewed clinical trials to assess the effectiveness of single time-point screening for AF, and reported that the proportion of patients with newly diagnosed AF was 1.4% in those aged ≥65 years, regardless of the screening setting [20]. The possible reasons for the different result in our study could be that physicians had a high level of awareness of stroke prevention because Akita Prefecture has the second highest age-adjusted death rate due to stroke among male patients and the seventh highest among female patients of all prefectures in Japan [21]. It is also possible that returning outpatients had already undergone diagnostic tests or treatment for AF but had not been formally diagnosed with AF. Thus, these factors were likely to decrease the influence of opportunistic AF screening.

To explore the possibility of a more appropriate evaluation of the effectiveness of opportunistic screening, an *ad-hoc* subgroup analysis was performed by focusing on the visit history before the observational periods. As anticipated, returning outpatients had notably more cardiovascular-related comorbidities compared with first-time outpatients, and returning outpatients may have undergone more thorough medical interventions, including cardiovascular-related treatment or diagnostic assessments, which may have affected the proportion of patients with newly diagnosed AF.

As a result, although the proportion of patients with newly diagnosed AF between the two observational periods remained the same (0.9%), among first-time outpatients, the proportion of patients with newly diagnosed AF was 1.6% in the pre-campaign period and slightly higher at 1.9% in the campaign period. In the present study, the greatest change in the proportion of patients with newly diagnosed AF was observed among patients aged 65–74 years (increase from 0.9% to 1.5%), suggesting that opportunistic AF screening may be a feasible option for the early diagnosis of AF in the younger population. The results were similar to those of a previous study by Fitzmaurice et al. [13]. Interestingly, among first-time outpatients aged 75–84 years in our study, the proportion of patients with newly diagnosed AF decreased from the pre-campaign period to the campaign period. The reason for this result is yet to be elucidated and further study may be needed. However, it could be speculated that a possible time lag between ECG and detection of an irregular pulse, which could occur when patients are referred to cardiologists, might lead to misdiagnosis due to the patient reverting to sinus rhythm; this factor may have affected the results. In addition, it is possible that the number of first-time patients in both study periods was too low to allow solid conclusions to be drawn.

Our original hypothesis was that opportunistic AF screening would benefit all outpatients without a history of AF aged ≥65 years in this study. However, returning outpatients who continued to visit the clinic and receive daily medical care were not likely to receive more benefits than expected, probably because they had already received some form of medical care for AF. Owing to the high proportion of returning outpatients, the results of the whole population did not change throughout the observational periods. However, the proportion of patients with newly diagnosed AF appeared to be higher among first-time outpatients during the campaign period. Our findings suggest that 1-year opportunistic AF screening may be a feasible screening strategy for first-time patients in the Japanese clinical setting. Implementation of opportunistic AF screening targeting first-time outpatients could be a more feasible option compared with opportunistic AF screening of all outpatients.

This study has several limitations. First, study clinics were not selected randomly; moreover, general practitioners who participated in the study did so on a voluntary basis. Therefore, this may have introduced selection bias and affected AF screening. Second, claims data were used to retrospectively identify patients with AF. Claims data, which are administrative data, do not include as specific or detailed clinical information as medical charts (e.g., quantifiable data such as pulse deficit). Therefore, patients with newly diagnosed AF identified by claims codes may not be matched completely with those identified by medical chart reviews. Third, physicians decided to participate during the pre-campaign period; therefore, physicians’ willingness to participate might have led to overestimation of the proportion of patients with newly diagnosed AF during the pre-campaign period. This also limits an analysis of the possible effect of the campaign itself on physicians’ behaviors. Forth, whether physicians regularly practice ECG after detecting an irregular pulse, regardless of the campaign, was not confirmed in this study, and this might have limited our analysis of the possible effect of encouraging physicians to perform opportunistic AF screening. Fifth, the accuracy of ECG interpretation for the definitive diagnosis of AF in this study depended on physicians’ experiences. However, it is likely that the deviation in accuracy among physicians was small and the diagnostic accuracy was high because primary care healthcare professionals in the primary care setting are generally competent in ECG interpretation. In addition, AF is a common disease, and in this study, if physicians had less experience in performing ECG, they had the opportunity to refer patients to hospitals for definitive diagnosis to ascertain the accuracy of the diagnosis. Sixth, it was difficult to identify the exact time lag between pulse check and ECG because of the characteristics of the claims data that were used in this study. Finally, due to the limited number of first-time patients, further studies will be needed for a more robust analysis.

## Conclusions

This is the first study to evaluate the feasibility and effectiveness of opportunistic AF screening for patients aged ≥65 years in primary care practice in Japan. The results suggest the feasibility of opportunistic AF screening in routine primary care practice in Japan. Of note, our findings suggest that 1-year opportunistic AF screening of first-time outpatients may be of clinical value.

## Supporting information

S1 Text Health insurance system in Japan and claims for medical insurance and the fee-for-service reimbursement system in Japan. (DOCX)

S2 Text Participating clinics in Daisen and Yokote. (DOCX)

S1 Table Proportion of patients with newly diagnosed AF during the pre-campaign and campaign periods among returning outpatients. (DOCX)

S1 Dataset SAS dataset. (SAS7BDAT)

S2 Dataset (SAS7BDAT)

S3 Dataset (XLSX)
