# Supplementary material for: Acceleration of opportunistic atrial fibrillation screening for elderly patients in routine primary care
Source: PLoS One. 2020 Dec 30;15(12):e0244240. doi: 10.1371/journal.pone.0244240 (PMC7773196; doi:10.1371/journal.pone.0244240)
Supplement: S1 Text — (DOCX) [file pone.0244240.s001.docx]

**S1 Text. Participating clinics in Daisen and Yokote.**

***Daisen City***

Kazuya Sasaki*, Sasaki Internal Medicine Clinic; Yoshiya Toyoshima*, Toyoshima Clinic; Michihiro Abe*, Ota Clinic; Noriaki Konishi*, Konishi Gastrointestinal Medicine Clinic; Sakiko Arai*, Arai Clinic; Masateru Goto*, Goto Internal Medicine Clinic; Yasukazu Kimura*, Kimura Internal Medicine Clinic and Yoshiyuki Osawa*, Osawa Gastrointestinal Medicine Clinic

***Yokote City***

Akira Takahashi*, Takahashi Internal Medicine Clinic; Tadashi Ogiwara, Ogiwara Internal Medicine Clinic; Takao Kumagai*, Kumagai Clinic; Syu Hashimoto*, Hashimoto Internal Medicine Clinic

* The Akita study group
