# Supplementary material for: Acceleration of opportunistic atrial fibrillation screening for elderly patients in routine primary care
Source: PLoS One. 2020 Dec 30;15(12):e0244240. doi: 10.1371/journal.pone.0244240 (PMC7773196; doi:10.1371/journal.pone.0244240)
Supplement: S2 Text — (DOCX) [file pone.0244240.s002.docx]

**S2 Text.** **Health insurance system in Japan and Claims for medical insurance and the fee-for-service reimbursement system in Japan.**

***Heath insurance system in Japan***

The health insurance system in Japan is based on two main components: “the universal health insurance system” and “free-access”. All Japanese residents are enrolled in one of these two components within the health insurance system depending on their age and occupation [1]: The Advanced Elderly Medical Service (for those aged ≥75 years) and Employee’s Health Insurance or National Health Insurance (for those aged <75 years). Therefore, all Japanese residents are eligible for medical treatment in healthcare facilities within the insurance system. The patient’s copayment generally ranges between 10% and 30% of the total cost of treatment depending on their age. The remaining portion of the fees is covered by the insurer. Costs are strictly controlled by a fee schedule set by the National Government, based on a fee-for-service system.

In the free access system, all Japanese residents can select medical care facilities such as hospitals or clinics by themselves and can also freely transfer between facilities. Therefore, Japanese residents can receive medical services from several facilities at the same fee even if they are referred to a specialist.

***Claims for medical insurance and the fee-for-service reimbursement system in Japan***

Claims data are administrative information used for the fee-for-service reimbursement system in Japan. Claims data include detailed information, such as codes and specific numbers or frequency, of the medical services received by all individuals. Claims data are recorded every month by each healthcare facility. These data are then sent to the examination and payment organization, a specialized agency in charge of claims payment from the health care facilities within the insurance system. Once claims are approved, they are sent to each insurer who then pays the healthcare facility for the service provided minus the patient’s co-payment [2].

**References**

1. Overview of Health Care Insurance System. Available from: <http://www.mhlw.go.jp/english/wp/wp-hw4/dl/health_and_medical_services/P26.pdf>

2. Example of Medical Fee Points. In: Conceptual Chart of Insured Medical Treatment. Available from: <http://www.mhlw.go.jp/english/wp/wp-hw4/dl/health_and_medical_services/P28.pdf>
