# Supplementary material for: Acceleration of opportunistic atrial fibrillation screening for elderly patients in routine primary care
Source: PLoS One. 2020 Dec 30;15(12):e0244240. doi: 10.1371/journal.pone.0244240 (PMC7773196; doi:10.1371/journal.pone.0244240)
Supplement: S1 Table — (DOCX) [file pone.0244240.s003.docx]

**S1 Table. Proportion of newly detected AF patients during the pre-campaign and campaign periods among the returning outpatients.**

|  | Proportion of newly detected AF patients (%) | |  |  |
| --- | --- | --- | --- | --- |
|  | Pre-campaign period | Campaign  period | Comparison difference (95% CI) | Comparison Rate  (95% CI) |
| Total | 0.7 (59/8201) | 0.7 (60/8661) | −0.0 (−0.3, 0.2) | 1.0 (0.7, 1.4) |
| Sex |  |  |  |  |
| Male | 0.8 (22/2900) | 0.8 (26/3094) | 0.1 (−0.4, 0.5) | 1.1 (0.6, 1.9) |
| Female | 0.7 (37/5301) | 0.6 (34/5567) | −0.1 (−0.4, 0.2) | 0.9 (0.6, 1.4) |
| Age, years |  |  |  |  |
| 65–74 | 0.4 (14/3180) | 0.2 (8/3499) | −0.2 (−0.5, 0.1) | 0.5 (0.2, 1.2) |
| 75–84 | 0.8 (27/3280) | 0.9 (29/3283) | 0.1 (−0.4, 0.5) | 1.1 (0.6, 1.8) |
| ≥85 | 1.0 (18/1741) | 1.2 (23/1879) | 0.2 (−0.5, 0.9) | 1.2 (0.6, 2.2) |
| Comorbidities |  |  |  |  |
| Congestive heart failure | 1.6 (29/1761) | 1.9 (36/1919) | 0.2 (−0.6, 1.1) | 1.1 (0.7, 1.8) |
| Hypertension | 0.8 (48/6005) | 0.9 (53/6234) | 0.1 (−0.3, 0.4) | 1.1 (0.7, 1.6) |
| Diabetes | 1.0 (30/3143) | 0.6 (20/3542) | −0.4 (−0.8, 0.0) | 0.6 (0.3, 1.0) |
| History of stroke/TIA | 0.9 (7/815) | 0.7 (6/845) | −0.1 (−1.0, 0.7) | 0.8 (0.3, 2.4) |
| Ischemic heart disease | 1.2 (19/1534) | 1.2 (22/1765) | 0.0 (−0.7, 0.8) | 1.0 (0.5, 1.9) |
| Any comorbidity | 0.8 (56/7015) | 0.8 (59/7390) | 0.0 (−0.3, 0.3) | 1.0 (0.7, 1.4) |

Abbreviations: AF, atrial fibrillation; CI, confidence interval; TIA, transient ischemic attack.
